# Supplementary material for: Evaluation of Volatile Organic Compounds Obtained from Breath and Feces to Detect Mycobacterium tuberculosis Complex in Wild Boar (Sus scrofa) in Doñana National Park, Spain
Source: Pathogens. 2020 May 2;9(5):346. doi: 10.3390/pathogens9050346 (PMC7281121; doi:10.3390/pathogens9050346)
Supplement: Supplementary file 1 [file pathogens-09-00346-s001.pdf]

Supplementary Materials

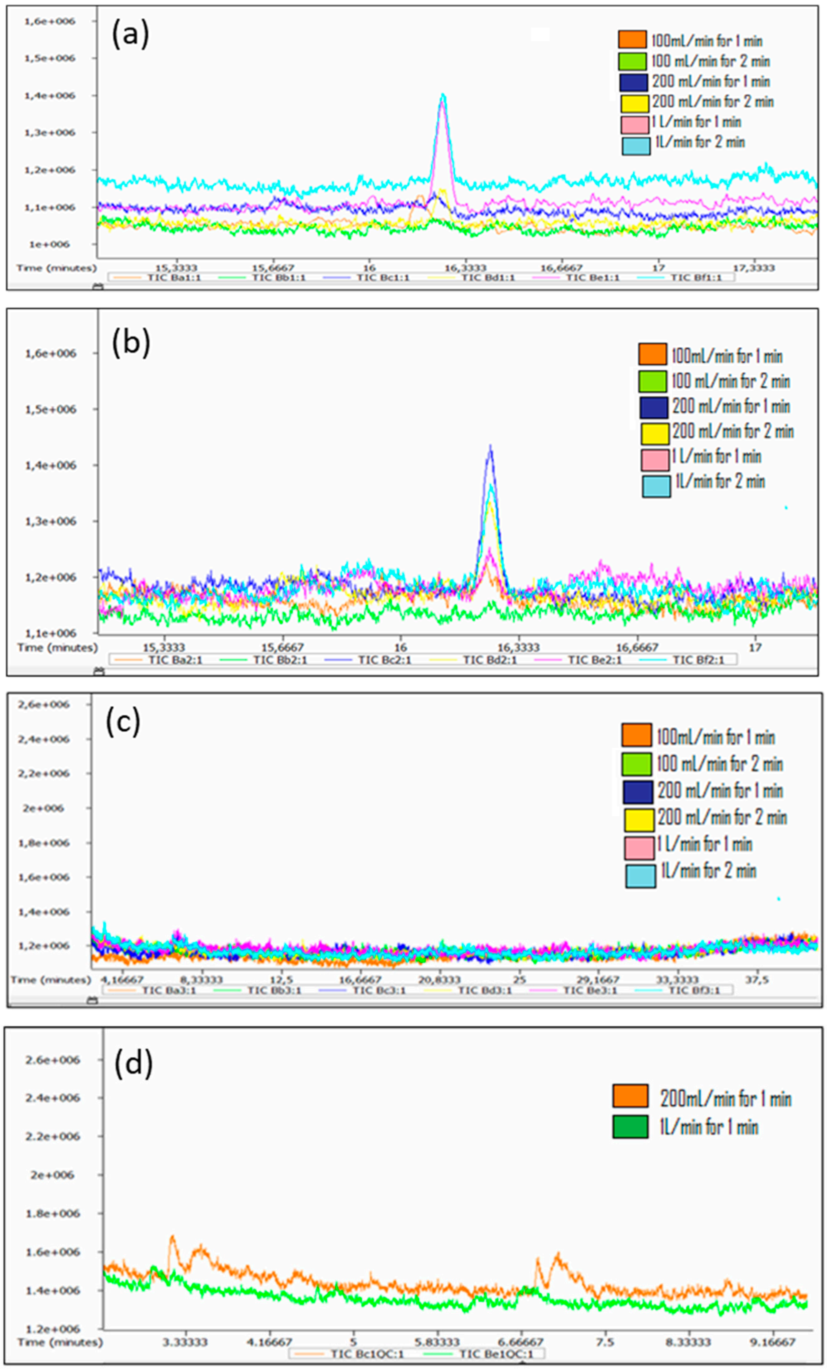

Figure S1. Most abundant chromatographic peaks of breath volatile organic compound (VOC) samples collected from one animal under six conditions over three days. (**a**) Most abundant chromatographic peak of the breath VOC samples collected on the first collection day; (**b**) most abundant chromatographic peak of the breath VOC samples collected on the second day; (**c**) chromatograms of the breath VOC samples collected on the third day, revealing no abundant chromatographic peak; (**d**) chromatograms of the quality control samples. Breath VOC samples were

collected from one animal over three days at a rate of 100 mL/min for 1 min, 100 mL/min for 2 min, 200 mL/min for 1 min, 200 mL/min for 2 min, 1 L/min for 1 min, and 1 L/min for 2 min.

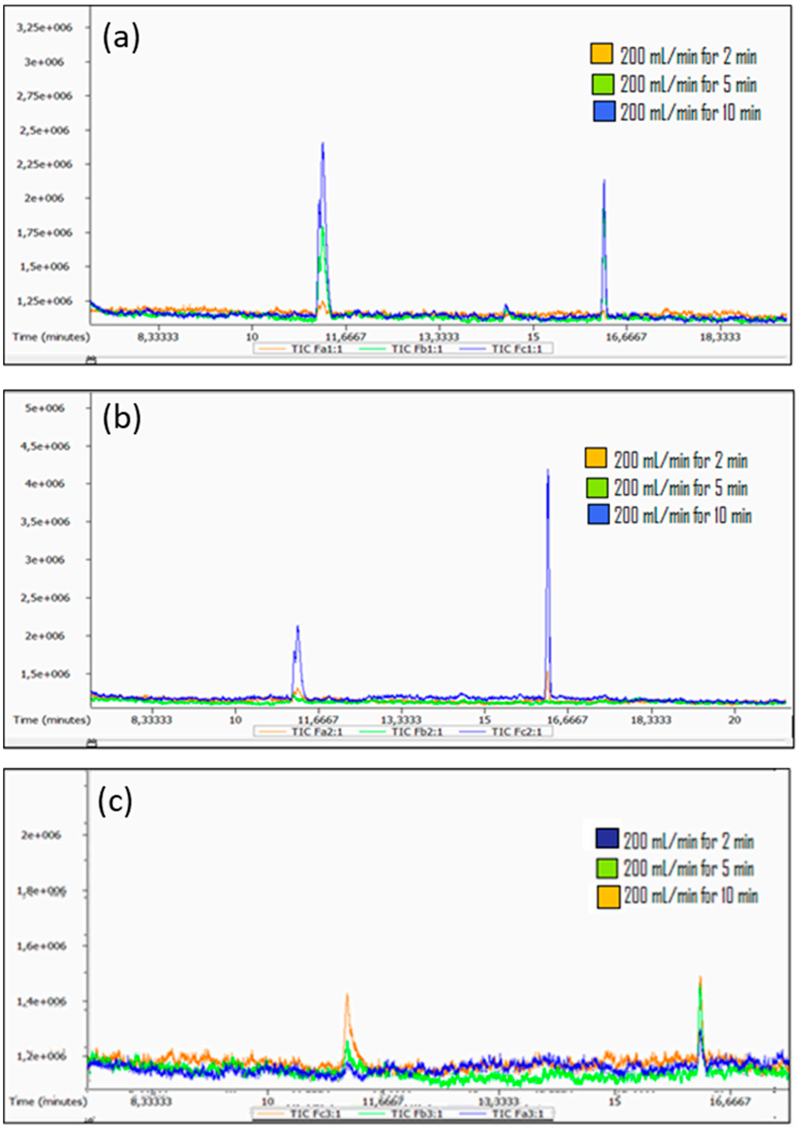

Figure S2. Most abundant chromatographic peaks of fecal volatile organic compound (VOC) samples collected from one animal under three conditions over three days. Most abundant chromatographic peaks of the fecal VOC samples collected during (**a**) the first collection day, (**b**) the second collection day, and (**c**) the third collection day. Fecal headspace air was collected at 200 L/min for 2 min, 200 L/min for 5 min, and 200 L/min for 10 min. Three samples were obtained under each condition.
